# Up-regulation of inflammation-related LncRNA-IL7R predicts poor clinical outcome in patients with cervical cancer

**DOI:** 10.1042/BSR20180483

**Published:** 2018-06-12

**Authors:** Yangyang Fan, Yan Nan, Juanjuan Huang, Hui Zhong, Weidong Zhou

**Affiliations:** 1 Department of Obstetrics, Shaanxi Provincial People's Hospital. Xi'an City, Shaanxi Province. 710068, Chinaa; 2 Department of Gynecology. Northwest Women and Children Hospital. Xi'an, Shaanxi Province, 716001, China; 3 Department of Obstetrics, affiliated Hospital of Yan'an University. Yan'an, Shaanxi Province, 716000, China; 4 Department of Gynecology, No. 215 Hospital of Shaanxi Nuclear Industry. Xianyang, Shaanxi Province, 712000, China; 5 Department of Obstetrics and Gynecology, The first Hospital of Yulin City. Shaanxi Province, 718000, China

**Keywords:** cervical cancer, Inflammation, Lnc-IL7R, prognosis

## Abstract

The long-term chronic inflammation of cervical intraepithelial neoplasia (CIN) induces the initiation and progression of cervical cancer. Long non-coding RNAs (LncRNAs) are being identified to be involved into inflammation and carcinogenesis and could function as cancer biomarkers in clinical. However, the significance of inflammation-related LncRNA (e.g. *LncRNA-IL7R*) in cervical cancer is limited. We, here, investigated the clinical role of inflammation-related *LncRNA-IL7R* (*Lnc-IL7R*) in healthy cervical tissue (*n*=15), CIN 1/2/3 (*n*=35), cervical cancer (*n*=70), and clarified its function via knockdown *in vitro* and *in vivo*. The results showed that the expression of *Lnc-IL7R* was increased from normal tissues to neoplastic lesions and cervical cancer. Up-regulated *Lnc-IL7R* positively correlated to tumor size, International Federation of Gynaecology and Obstetrics (FIGO) stage, and lymph node metastasis (LNM). Patients with high expression of *Lnc-IL7R* had poor prognosis with short overall survival (OS) time, and Cox regression analysis revealed that *Lnc-IL7R* could be independent prognostic factor for cervical cancer. Moreover, knockdown of *Lnc-IL7R* by two different siRNAs in cervical cancer cell lines Hela and SiHa induced impaired cell vitality and caspase-3-dependent apoptosis *in vitro*. Furthermore, inhibition of *Lnc-IL7R in vivo* significantly restricted the tumor growth with decreased expressions of proliferation index Ki-67 and *Lnc-IL7R*. These data indicated that *Lnc-IL7R* predicts a poor clinical outcome of cervical cancer patients, and knockdown of *Lnc-IL7R* is amenable to the treatment of cervical cancer.

## Introduction

Cervical cancer is the major cause of death from gynecological cancers and is the third most common malignancy in women worldwide with a global incidence of 500000 and mortality of 250000. More than 85% of these cases and deaths occurred in developing countries, including China [1,2]. Persistent infection with oncogenic subtypes of the human papillomavirus (HPV) results in chronic inflammation, leading to the cervical intraepithelial neoplasia (CIN) and carcinogenesis of uterine cervix [3]. The signs and symptoms of cervical cancer often occur in the later stages of the infection (CIN 1, 2, and 3), thus, the detection of tumorigenesis at the microscopic level is inefficient in earliest stages of diagnosis [4]. Currently, some proteins and HPV DNA-based biomarkers are developed for the diagnosis of cervical cancer in clinical, such as SSC-Ag, CA-125, CEA, and Cytokeratins [4]. In addition, no single screening method exists that is highly sensitive, highly specific, affordable, and practical [5]. Therefore, it is still urgent to identify new and effective prognostic markers and therapeutic strategies to improve treatment of cervical cancer.

The majority of transcribed RNAs are non-coding in mammalian cells, which do not contain protein-coding sequences. These transcripts are eventually, on one hand, processed into small RNAs including miRNAs, Piwi-interacting RNAs (piRNAs), tRNA-derived stress-induced fragment RNAs, and small nucleolar RNAs (snoRNAs), and, on the other hand, processed into long non-coding RNAs (LncRNAs) [6,7]. The miRNAs and LncRNAs have demonstrated to be involved into carcinogenesis and functioned as diagnostic and prognostic biomarkers [8,9]. *miR-138* expression in cervical cancer cells is significantly lower than that in normal tissues, which causes telomerase activation and carcinogenesis [10]. Let-7b, let-7c, *miR-23b, miR-143*, and *miR-196b* were down-regulated in cell lines and tumor tissue compared with normal tissue whereas *miR-21* was up-regulated [10]. Up-regulated LncRNA HOTAIR in cervical cancer tissues and correlated with International Federation of Gynaecology and Obstetrics (FIGO) stage, lymphatic metastasis, size of tumor in cervical cancer progression and could be a potential target for diagnosis as well as an independent predictor for overall survival (OS) [11]. The tumor-suppressor LncRNA GAS5 was down-regulated in cervical cancer tissues, significantly correlated to advanced cancer progression, and identified as a biomarker for forecasting the clinical states of patients in cervical cancer [12]. Similarly, LncRNA *MALAT1* and HOTAIR were reported to predict the poor clinical outcome of patients with cervical cancer [9]. Chronic inflammation is essential for the development of cervical cancer, but the role of inflammation-related LncRNA in cervical is unclear.

Cui et al. [13] found an inflammation-regulated *LncRNA-IL7R* (*Lnc-IL7R*) was capable of diminishing the LPS-induced inflammatory response, inhibiting the expressions of LPS-induced E-selectin, VCAM-1, IL-6, and, IL-8. Ding et al. [14] reported that *Lnc-IL7R* was also induced in response to TLR3 stimulation and negatively regulated the TNF-α and IL-8. *Lnc-IL7R* has been found to participate in multiple sclerosis, chemotherapy, and acute respiratory distress syndrome (ARDS) [14–16]. The *Lnc-IL7R* levels were correlated with the severity of ARDS and could predict 28-day mortality in the patient’s cohort [15]. In the present study, we investigated the potential clinical role of *Lnc-IL7R* in cervical cancer. The expression of *Lnc-IL7R* in normal, CIN, and cervical cancer samples, and its correlation to clinical characteristics were analyzed. The functions of *Lnc-IL7R in vitro* and *in vivo* were also assessed in cervical cancer cell lines Hela and SiHa.

## Materials and methods

### Patients and tissue specimens

Healthy cervical tissue (*n*=15), CIN 1/2/3 (*n*=35), cervical cancer (*n*=70) were collected to determine the expressions of *Lnc-IL7R* and TNF-α, and median age was 35, 37, and 51 years, respectively. The sample collection in the present study was approved by Yan’an People’s Hospital and all patients completed informed consent forms and the healthy individuals’ recruitment were also obtained from Yan’an People’s Hospital. All these retrospective specimens were handled and anonymized according to ethical and legal standards. The tissue samples were isolated from surgical removal and then stored at −80°C until use.

The patients with primary cervical cancer were diagnosed by Hematoxylin and Eosin staining by experienced pathologists from Department of Pathology at Yan’an People’s Hospital. None of the patients underwent preoperative chemotherapy and/or radiotherapy. Patients with other kinds of cancer or some autoimmune disease (e.g. rheumatoid arthritis, systemic lupus erythematous, diabetes etc.) were absolutely excluded. Besides, pregnant and lactating individuals were also excluded from the present study. The 70 patients were followed up until 1 October 2017. The high or low expressions of *Lnc-IL7R* were defined by the median of the expression. The three grades of *Lnc-IL7R* expression were defined as Grade 1 (25th percentile), Grade 2 (25–50th percentile), and Grade 3 (>50th percentile).

### RNA isolation and quantitative real-time RT-PCR

Total RNA was extracted using TRIzol reagent (Invitrogen) according to standard RNA isolation protocol. The conditions of reverse-transcription for cDNA with Reverse Transcription System were 42°C for 5 min, 99°C for 20 min, 4°C for 5 min. The SYBR Green PCR Master Mix (Applied Biosystems), according to the manufacturer’s instructions, was performed for quantitative real-time RT-PCR (qRT-PCR) and 2^−ΔΔ*C*_T_^ method was used to estimate relative expression changes in genes including *Lnc-IL7R* and *TNF-α*. The expression levels were normalized to *GAPDH* for gene expression.

### Cell lines and reagents

The cervical cancer cell lines Hela and SiHa were purchased from the cell bank of the Chinese Academy of Sciences (Shanghai, China) and cultured in Dulbecco’s modified Eagle’s medium (DMEM) supplemented with 10% FBS (Life Technologies, U.S.A.), ampicillin and streptomycin at 37°C, 5% CO_2_ conditions. *siRNA-Lnc-IL7R* or negative control was purchased from RiboBio (Guangzhou, China). Anti-GAPDH, Ki-67, caspase-3, and Bcl-2 antibodies were obtained from Cell Signaling Technology (Denver, MA) and Abcam (U.S.A.).

### Cell transfection

The Hela and SiHa cell lines were cultured to ~80% confluence in 12/96-well plates and then, using Lipofectamine 2000 (Invitrogen, U.S.A.), the cells were transfected with indicated agents according to the manufacturer’s instructions. After transfection for the indicated time, the cells were harvested for further experiments.

### CCK-8 assay

The Hela and SiHa cell lines were transfected with siRNAs and then were harvested to wash with PBS, and then cell counting kit-8 (Kumamoto, Japan) mixed with DMEM was used for cell viability assay, and the absorbance was measured at 450 nm by a microplate reader.

### Hoechst staining assay

The conditional Hela and SiHa cell lines were harvested and incubated with Hoechst 33342 (5 μg/ml, Sigma, U.S.A.) for 10 min at room temperature. Following washing with 0.5% Triton X-100 in PBS, the changes in nuclear morphology were observed under a fluorescence microscope (Olympus, Tokyo, Japan). Each experiment was performed in triplicate and repeated three times.

### Western blot

According to the manufacturer’s protocol, cells for Western blot were collected and total protein was isolated from the cell samples. Detailed procedures for immunoblotting are described elsewhere [17]. GAPDH was used as the loading control in the Western blotting.

### Immunohistochemistry

To determine the expression of Ki-67 in tissues, 2-μm thick, formalin-fixed, and paraffin-embedded specimen sections were used. After the slides were incubated in xylene for 5 min, 100% ethanol was used for 10 min, 95% ethanol for 10 min. Antigen unmasking was performed and then the slides were blocked with 3% hydrogen peroxide for 30 min at room temperature. Then the primary antibody for Ki-67 was incubated the FFPE specimen sections at 4°C overnight, then the biotinylated horse secondary antibody and streptavidin-horseradish peroxidase (Zymed Laboratories Inc.) were used for the detection of Ki-67. After that, the EnVision Detection System kit (DAKO, Denmark) was used for the DAB chromogen followed by nuclear staining using Hematoxylin.

### Tumor model

To investigate the role of *Lnc-IL7R in vivo*, Hela cells were transfected with lentivirus vector of *siRNA-Lnc-IL7R* or negative control, 2 × 10^6^ Hela cells were subcutaneously injected in rear flank of nude mice (five per group). The tumor sizes were measured 3 days apart and the tumor volumes were calculated: V (cm^3^) = width^2^ (cm^2^) * length (cm)/2. On day 2, the mice were killed.

### Statistical analyses

The results were analyzed by the Statistical Package for Social Sciences version 16.0 (SPSS 16.0, SPSS Inc., Chicago, IL, U.S.A.) and the Prism statistical software package (version 5.0, GraphPad Software Inc.). Kolmogorov–Smirnov and Shapiro–Wilk tests showed that the expression of *Lnc-IL7R* in each group did not follow a normal distribution. The Mann–Whitney U-test was used to compare the two groups (e.g. normal compared with CIN) and the differences between more than two groups (e.g. CIN1/2/3) were analyzed by the Kruskal–Wallis test. Kaplan–Meier survival curves and the log-rank statistic were used to analyze the prognostic significance of *Lnc-IL7R*. Correlations between the expression of *Lnc-IL7R* and *TNF-α* were analyzed by Spearman’s Rho analysis. Correlations of expression of *Lnc-IL7R* and clinicopathological characters were analyzed by Pearson chi-square. Cox proportional hazards regression was used for univariate and multivariate analysis of OS according to *Lnc-IL7R* expression. *P*<0.05 was considered statistically significant. All experiments were performed at least three times.

## Results

### The expression of *Lnc-IL7R* is increased during the development of cervical cancer

**Figure 1:**
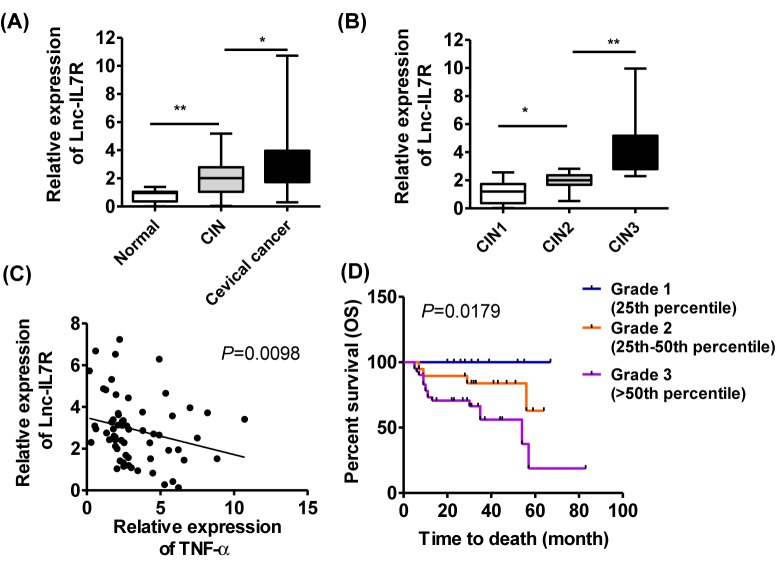
Up-regulated *Lnc-IL7R* predicts poor clinical outcome in cervical cancer (**A**,**B**) The expression of *Lnc-IL7R* in normal cervix (*n*=15), (CIN 1/2/3) (*n*=35), and cervical cancer samples (*n*=70) tissue samples were determined by Q-PCR. (**C**) The correlation between *Lnc-IL7R* and *TNF-α* was analyzed. (**D**) The OS time was analyzed by Kaplan–Meier survival curves according to the three degrees of *Lnc-IL7R*. *P<0.05, **P<0.01, data represent the means ± S.D.

To investigate the expression pattern of *Lnc-IL7R* in cervical cancer, normal cervix (*n*=15), (CIN1/2/3) (*n*=35), and cervical cancer samples (*n*=70) were collected and the results of Q-PCR indicated that the expression of *Lnc-IL7R* was increased during the development of cervical cancer. The cancer tissues harbored the highest *Lnc-IL7R* level (Figure 1A). We further analyzed the expression of *Lnc-IL7R* in different groups, 12 in 15 (80%) of normal cervix showed the low expression of *Lnc-IL7R* and only 3 in 15 (20%) showed the high expression of *Lnc-IL7R*. But in the CIN samples, the high expression of *Lnc-IL7R* accounted for (57%) 20 in 35, which was further increased to (59%) 41 in 70 in cervical cancer (Table 1).

**Table 1:** *Lnc-IL7R* expression in patients with cervical cancer

Types	*n*	*Lnc-IL7R* expression		*χ^2^*	*P*
		Low (%)	High (%)		
Normal cervix	15	12 (80.0)	3 (20.0)	7.672	0.02*
CIN	35	15 (42.8)	20 (57.2)		
Cervical cancer	70	29 (41.4)	41 (58.6)		

*P<0.05, statistically significant, *χ^2^* represents Pearson chi-square value.

Moreover, the *Lnc-IL7R* levels in different stages of CIN were determined. The results indicated the similar trend that high stage of CIN correlated to high *Lnc-IL7R* level in tissue samples (Figure 1B) and 83% CIN3 tissues harbor high expression of *Lnc-IL7R* (Table 2).

**Table 2:** The *Lnc-IL7R* expression in patients with CIN

CIN speicemen	*n*	*Lnc-IL7R* expression		*χ2*	*P*
		Low (%)	High (%)		
CIN1	13	9 (69.2)	4 (30.8)	7.087	0.029*
CIN2	10	4 (40)	6 (60)		
CIN3	12	2 (16.7)	10 (83.3)		

*P<0.05, statistically significant, *χ^2^* represents Pearson chi-square value.

Because *TNF-α* as an antitumor factor is negatively regulated by *Lnc-IL7R* [14], thus, we investigated its correlation in cervical cancer. We found that higher *Lnc-IL7R* level corresponded to lower *TNF-α* level in tumor tissues (Figure 1C). The *Lnc-IL7R*-induced decreased expression of *TNF-α* might be conducive to the progression of cervical cancer.

### Up-regulated expression of *Lnc-IL7R* correlates to poor clinical outcome in cervical cancer

Since the *Lnc-IL7R* increased with the progression of cervical cancer, we next estimated the correlation between the expression of *Lnc-IL7R* and the clinicopathological characteristics of cervical patients. As shown in Table 3, the expression of *Lnc-IL7R* had no association with age, histology, differentiation, vascular invasion, and the HPV status. But patients with higher tumor size, FIGO stage, and lymph node metastasis (LNM) have more high-expressed *Lnc-IL7R*. These data implicated that, in patients with cervical cancer, *Lnc-IL7R* could predict a poor clinical outcome including tumor size, FIGO, and LNM.

**Table 3:** Relationships between *Lnc-IL7R* expression and clinicalpathologic characteristics in cervical cancer patients

Characteristics	*n* (=70)	*Lnc-IL7R* expression		*P*
		Low (*n*/%)	High (*n*/%)	
**Age**				
<50	31	14 (45.2)	17 (54.8)	0.572
≥50	39	15 (37.1)	24 (62.9)	
**Tumor size (cm)**				
≤4	45	23 (51.1)	22 (48.9)	0.027*
>4	25	6 (24)	19 (76)	
**Histology**				
Squamous	52	24 (46.2)	28 (53.8)	0.173
Adenocarcinoma	18	5 (27.8)	13 (72.2)	
**FIGO stage**				
I–II	39	21 (53.8)	18 (46.2)	0.018*
III–IV	31	8 (25.8)	23 (74.2)	
**Differentiation**				
Well	26	12 (46.2)	14 (53.8)	0.537
Moderate to poor	44	17 (38.6)	27 (61.4)	
**LNM**				
No	49	25 (51)	24 (49)	0.013*
Yes	21	4 (19)	17 (81)	
**Vascular invasion**				
No	46	20 (43.5)	26 (56.5)	0.63
Yes	24	9 (37.5)	15 (62.5)	
**HPV**				
Negative	30	14 (46.7)	16 (43.3)	0.441
Positive	40	15 (37.5)	25 (62.5)	
**Total**	70	29 (100)	41 (100)	

*P<0.05, statistically significant.

### *Lnc-IL7R* is an independent factor for cervical cancer

The prognostic role of *Lnc-IL7R* was also investigated in the present study. The 70 patients were followed up until 1 October 2017. The OS time was analyzed according to the expression of *Lnc-IL7R*, which indicated that patients with highly expressed *Lnc-IL7R* had shorter OS than those with lowly expressed *Lnc-IL7R* (chi square = 5.605 by log-rank test) (Figure 1D).

Univariate and multivariate analyses were performed, the results revealed that age, histology, FIGO, LNM, vascular invasion, and differentiation were not independent prognostic indicators for OS, but the tumor size, the HPV status, and the expression of *Lnc-IL7R* were the independent prognostic factors for the OS of patients with cervical cancer (Table 4).

**Table 4:** Prognostic factors in the Cox proportional hazards model

Variables	OS					
	HR	Univariate 95% CI	Sig.	HR	Multivariate 95% CI	Sig.
**Age**						
<50 compared with ≥50	1.125	0.827–2.491	0.736			
**Tumor size (cm)**						
<4 compared with ≥4	2.435	1.730–3.440	0.008*	3.837	2.312–6.384	0.0001*
**Histology**						
Squamous compared with adenocarcinoma	0.837	0.842–2.391	0.721			
**FIGO stage**						
I–II compared with III–IV	1.923	1.723–3.342	0.023*	3.128	3.023–4.298	0.127
**Differentiation**						
Low compared with moderate-high	1.182	0.728–1.942	0.732			
**LNM**						
– compared with +	2.442	1.401–3.894	0.013*	4.104	1.202–3.390	0.073
**Vascular invasion**						
No compared with high	1.879	1.237–2.823	0.026*	2.923	1.949–5.127	0.533
**HPV**						
Negative compared with positive	0.394	0.122–0.739	0.011*	0.418	0.232–0.823	0.027*
***Lnc-IL7R* expression**						
Low compared with high	3.392	1.750–5.979	0.008*	5.826	2.213–7.129	0.0001*

Abbreviations: CI, confidence interval; HR, hazard ratio; Sig, significance. *P<0.05, statistically significant.

### Knockdown of *Lnc-IL7R* induces caspase-3 dependent apoptosis *in vitro*

**Figure 2:**
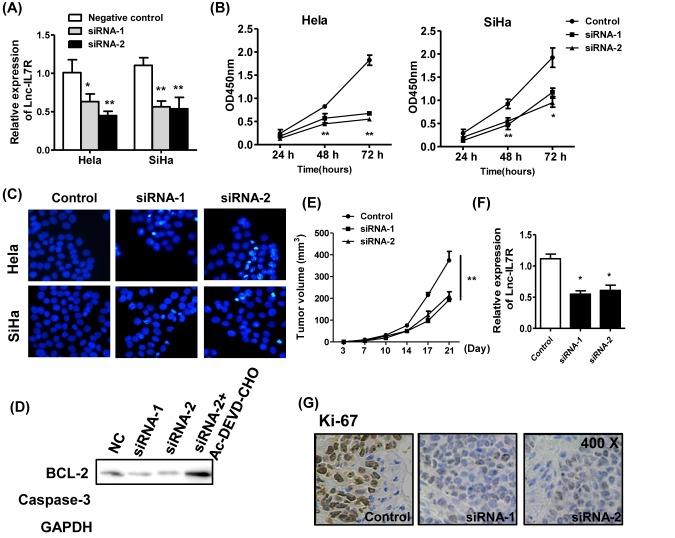
Knockdown of *Lnc-IL7R* inhibits the tumor growth of cervical cancer (**A**) The efficiency of knockdown in two cervical cancer cell lines Hela and SiHa was determined. (**B**,**C**) The cell vitalities and apoptosis of two cell lines were estimated by CCK-8 and Hoechst. (**D**) The expressions of BCL-2 and caspase-3 were assessed by western blot. (**E**) The mean tumor size (mm^3^) was analyzed. (**F**,**G**) The expressions of *Lnc-IL7R* and Ki-67 proliferation index were estimated by Q-PCR and immunohistochemistry. *P<0.05,**P<0.01.

To provide the potential mechanism of the tumorigenic role of *Lnc-IL7R*, two cervical cancer cell lines Hela and SiHa were used. Knockdown of *Lnc-IL7R* by two independent siRNA in two cell lines effectively decreased the *Lnc-IL7R* level (Figure 2A). The cell vitalities were analyzed and found that knockdown of *Lnc-IL7R* significantly inhibited the cell vitalities of both the cell lines (Figure 2B). The apoptosis of two cell lines were also found to be induced by the *siRNA- Lnc-IL7R* (Figure 2C).

In addition, the apoptosis-related molecules were determined by WB. The anti-apoptosis factor BCL-2 was down-regulated by the knockdown of *Lnc-IL7R* and the caspase-3 was also cleaved in response to the knockdown of *Lnc-IL7R*. This process could be restored by the treatment of caspase-3 inhibitor Ac-DEVD-CHO (25 μmol/l) (Figure 2D).

### *Lnc-IL7R* inhibition *in vivo* restricts tumor growth

Given the tumorigenic role of *Lnc-IL7R in vitro*, we next estimated the efficiency of *Lnc-IL7R* knockdown for tumor progression by lentivirus vector of *siRNA-Lnc-IL7R in vivo*. The xenograft model of human HeLa was established. The results showed that the inhibition of *Lnc-IL7R* could effectively inhibit tumor growth (Figure 2E), which might be related to decreased expression of *Lnc-IL7R* (Figure 2F). The expression of tumor proliferation indication Ki-67 was also inhibited by knockdown of *Lnc-IL7R in vivo* (Figure 2G).

## Discussion

In some low-income countries, cervical cancer is the most common cancer in women. Therefore, cervical cancer is a public health problem worldwide [5]. The treatment strategy for cervical cancer depends on the clinical stage, which is defined by the FIGO staging system. Traditional clinicopathological characteristics are not sufficiently reliable for predicting clinical outcomes or for guiding optimal treatment strategies [18]. In the present study, an inflammation-related LncRNA, *Lnc-IL7R*, was up-regulated with the initiation and development of cervical cancer, which positively correlated to the tumor size, FIGO stage, and LNM, and could predict the poor prognosis of patients with cervical cancer.

The non-coding RNAs (ncRNAs) participate in the post-transcription of gene expressions or interact with proteins to regulate the target mRNAs and proteins that were involved into carcinogenesis [19]. Amounting evidences identified the clinical diagnostic and prognostic role of ncRNAs. Using an established PCR-based miRNA assay to analyze 102 cervical cancer samples, Hu et al. [20] identified *miR-200a* and *miR-9* that could predict poor patient survival and patients with high *miR-200a* and *miR-9* had shorter survival time. Another study in small cell carcinoma of the cervix (SCCC) demonstrates that down-regulation of seven miRNAs (e.g. let-7c, *miR-100, miR-125b*) associated with advanced-stage SCCC patients (FIGO IB2-IV) compared with early-stage SCCC patients (FIGO IB1), six miRNAs with metastasis, and two with poor prognosis (e.g. *miR-100, miR-125b*) [21]. Yang et al. [22] reported that the expression of oncogene *LncRNA-MALAT1* was significantly increased in cervical cancer than in normal tissues and correlated with tumor size, FIGO stage, vascular invasion, and LNM and is an independent predictor for OS of cervical cancer. *LncRNA CCAT2* was also found to be up-regulated in cervical squamous cell cancer tissues, patients with high expression of lncRNA CCAT2 had poor OS and PFS rates and was an independent poor prognostic factor for cervical cancer patients [23]. In the present study, we found the clinical significance of *Lnc-IL7R* in cervical cancer, the expression of *Lnc-IL7R* was elevated in CIN tissues and cervical cancers, which predicted the poor clinical outcome of patients and could be an independent factor for cervical cancer. This finding suggested that the inflammation-related *Lnc-IL7R* functioned as an oncogene in cervical cancer.

The potential mechanisms of LncRNA in cervical cancer had been reported. Kim et al. [11] found that knockdown of HOTAIR in cervical cancer cell lines inhibited cell proliferation, migration, and invasion via the regulation of epithelial-to-mesenchymal transition (EMT)-related genes. We here found that knockdown of Lnc-IL7R significantly impaired the cell vitalities of two cervical cancer cell lines HeLa and SiHa. The apoptosis was also induced by the Lnc-IL7R inhibition by reduced expressions of BCL-2 and caspase-3, which could be restored by the caspase-3 inhibitor. CCHE1 overexpression promoted the proliferation of cervical cancer cell. RNA pull-down assays confirmed that CCHE1 physically associated with proliferating cell nuclear antigen (PCNA) to enhance the expression of PCNA [24]. The tumorigenic role of LncRNA in cervical cancer *in vivo* was limited. Using the siHOXA11-AS-transfected HeLa cells revealed that HOXA11-AS strongly induced tumor growth in xenograft experiments with the decreased cancer stemness and triggered the EMT program [25]. We found that the administration of siRNA for Lnc-IL7R could inhibit the tumor growth *in vivo* and decrease the expression of Ki67.

## Conclusion

We reported that an oncogene *Lnc-IL7R* is increased during the development of cervical cancer, and could be an independent factor for the patients with cervical cancer. Knockdown of *Lnc-IL7R* inhibited the tumor growth *in vivo*, which could be a potential treatment for cervical cancer.

## Abbreviations

ARDS: acute respiratory distress syndrome
CIN: cervical intraepithelial neoplasia
DMEM: Dulbecco’s modified Eagle’s medium
EMT: epithelial-to-mesenchymal transition
FIGO: International Federation of Gynaecology and Obstetrics
HPV: human papillomavirus
LncRNA: long non-coding RNA
LNM: lymph node metastasis
OS: overall survival
PCNA: proliferating cell nuclear antigen
SCCC: small cell carcinoma of the cervix
